# Characteristics of CVA16 B1c strains isolated for the first time in the Heilongjiang Province of China

**DOI:** 10.3389/fmicb.2025.1634547

**Published:** 2025-08-06

**Authors:** Tingting Yang, Qiang Sun, Hui Liu, Ming Yang, Shuhong Chen, Wei Sun, Jun Xu, Dongmei Yan, Shuangli Zhu, Zhenzhi Han, Jinbo Xiao, Huanhuan Lu, Ying Liu, Qian Yang, Yong Zhang

**Affiliations:** ^1^Jinan Center for Disease Control and Prevention, Jinan, China; ^2^National Key Laboratory of Intelligent Tracking and Forecasting for Infectious Diseases (NITFID), National Institute for Viral Disease Control and Prevention, Chinese Center for Disease Control and Prevention, Beijing, China; ^3^National Polio Laboratory, World Health Organization Polio Reference Laboratory for the Western Pacific Region, National Institute for Viral Disease Control and Prevention, Chinese Center for Disease Control and Prevention, Beijing, China; ^4^National Health Commission Key Laboratory of Laboratory Biosafety, National Institute for Viral Disease Control and Prevention, Chinese Center for Disease Control and Prevention, Beijing, China; ^5^Heilongjiang Provincial Center for Disease Control and Prevention, Harbin, China; ^6^Laboratory of Virology, Beijing Key Laboratory of Etiology of Viral Diseases in Children, Capital Institute of Pediatrics, Beijing, China

**Keywords:** CVA16 B1c, epidemiology, evolutionary origin analysis, recombination, enteroviruses

## Abstract

Hand, foot, and mouth disease (HFMD) represents a globally prevalent infectious disease that is caused by enteroviruses. Enterovirus A71 (EV-A71), coxsackievirus A16 (CVA16), and coxsackievirus A6 (CVA6) are recognized as the predominant causative agents of HFMD. CVA16 is a member of the genus *Enterovirus* within the family *Picornaviridae*. B1a and B1b are the most prevalent subgenotypes, whereas the B1c subgenotype is relatively scarce. In this study, a comprehensive analysis was conducted on 15 of CVA16 B1c strains isolated from samples of patients diagnosed with HFMD in Jixi (Heilongjiang Province, China) in 2022. Subsequently, whole genome sequencing of these strains was carried out. Phylogenetic origin and potential recombination events were analyzed by aligning sequences of isolated of CVA16 B1c strains with related sequences deposited in GenBank. The CVA16 B1c isolates examined in this study exhibited a high degree of similarities. Specifically, the nucleotide similarity within the *VP1* region ranged from 99.6 to 100%. The average nucleotide substitution rate of CVA16 B1c viruses worldwide was estimated to be 5.14 × 10^−3^ (4.13–6.27 × 10^−3^) substitution/site/year, and the most recent common ancestor could be traced back to 2003. The earliest CVA16 B1c strain isolated in China was traced back to 2011. Transmission pathway analysis suggested that Chinese strains may have originated in India. Recombination analysis showed that CVA16 B1c strains likely undergone recombination events with EV-A71 and CVA4. In conclusion, the analysis of a cluster of CVA16 B1c cases detected for the first time in Heilongjiang Province not only expanded the gene sequence library of CVA16 B1c strains in China but also offered an epidemiological basis for further investigations into the antigen–antibody interactions and pathogenicity of CVA16 B1c.

## 1 Introduction

Hand, foot and mouth disease (HFMD) is an infectious disease caused by various enteroviruses. It typically presents with mouth ulcers and rash on the hands and feet (Zhu et al., 2023). In 1957, an illness caused by coxsackievirus (mainly CVA16) was first reported in Toronto. Fever and mouth ulcers were characteristic manifestations of HFMD (Robinson et al., 1958). Previous studies have identified EV-A71 and CVA16 as the major causative pathogens of HFMD, which caused large-scale outbreaks in the United States and Asia–Pacific region (Zhu et al., 2023; González-Sanz et al., 2019; Noisumdaeng et al., 2019; Liu et al., 2000; Bible et al., 2008). CVA16 was first isolated in South Africa in 1951. According to the phylogenetic tree and genetic diversity of the *VP1* gene, CVA16 strains can be divided into three genotypes: A, B, and D (Hu et al., 2021). Gene group B viruses can be further divided into B1a, B1b, B1c, and B2 clades (Zhang et al., 2010a). B2 was the dominant clade before 2000, but B1 has gradually replaced B2 as the most common clade after 1997 (Perera et al., 2007). Genotype D was first identified in Peru in 2009 and then spread to France from 2011 to 2014 (Hassel et al., 2017). In 2016, the SH-HP-16-51 strain was isolated in a patient with mild HFMD in Shanghai, China, and tested positive for the D gene (Wang et al., 2018a).

In 2005, the CVA16 B1c subtype was isolated in Malaysia for the first time. In recent years, CVA16 B1c has been detected successively in Malaysia (Chen et al., 2013; Perera et al., 2007; Chan et al., 2012), Japan (Mizuta et al., 2013), India (Ganorkar et al., 2017; Mamidi et al., 2024; Rao et al., 2017; Palani et al., 2016), and other countries. Among them, the HFMD study that broke out in the Andaman Islands of India in 2013 showed that CVA16 was the main pathogenic factor and was closely related to the B1c gene group (Palani et al., 2016). In China, CVA16 B1a and B1b strains co-circulated and spread continuously (Liu et al., 2000; Zhang et al., 2010a; Han et al., 2020). However, B1c was only detected in Shanghai in 2014 (Wang et al., 2018b), Xinjiang in 2017, Beijing in 2016 and 2022 (Hu et al., 2021; Liang et al., 2024), Guangdong in 2018 and 2023 (Xie et al., 2020; Zeng et al., 2025), lacking systematic epidemiological analysis. In this study, CVA16 B1c isolates detected in Jixi (Heilongjiang Province, China) in 2022 were analyzed using molecular epidemiology approaches. The evolution and propagation origins of B1c strains in China were explored using comparisons with similar sequences in GenBank. These results enrich the CVA16 B1c gene database in China and provide scientific data for further prevention and control of infections caused by these viruses.

## 2 Materials and methods

### 2.1 Ethics statement

All throat swab samples collected in this study were sent to the national laboratory after being processed at provincial and municipal levels. All specimens were collected after informed consent was obtained. The experimental protocol was approved by the Second Ethics Review Committee of the National Institute for Viral Disease Control and Prevention at the Chinese Center for Disease Control and Prevention.

### 2.2 Sample collection and virus isolation

In 2008, China established a national HFMD pathogen surveillance network. Representative samples from each province are sent to the national HFMD laboratory for verification. Clinical cases of HFMD are diagnosed according to the Guidelines for the Diagnosis and Treatment of HFMD (2018 edition) (Li et al., 2018). In this study, isolates from throat swab samples of children with mild HFMD, originally sent from the Heilongjiang Province in 2022, were used. Samples were collected from Jixi (*n* = 46), Harbin (*n* = 5), Jiamusi (*n* = 6), Yichun (*n* = 3), Qiqihar (*n* = 12), and Mudanjiang (*n* = 1). The samples were treated and inoculated into human rhabdomyosarcoma cells. The infected viral suspension was harvested after a complete cytopathic effect was observed. The samples were transported to the National Polio Laboratory for further isolation and identification within 24 h according to a rigorous cold chain procedure. The *VP1* sequence was amplified using the universal EV primers. The genotype was determined using a criterion of *VP1* nucleotide similarity >75% (amino acid similarity >85%) (Oberste et al., 1999; Oberste and Pallansch, 2005). MEGA7.0 software (v7.0.25; Sudhir Kumar, Arizona State University, Tempe, AZ, USA) (Kumar et al., 2016) was used to compare *VP1* sequences, construct the phylogenetic tree through the “Models” option and reference sequence, and set the bootstrap value to 1,000 for the tree test. The gene subtype was determined based on the aggregation of the reference sequences in the evolutionary tree.

### 2.3 CVA16 B1c whole genome sequencing

The complete genome sequence was obtained using a “primer walk” strategy. 0001S48 was used as the upstream primer to amplify the start 5′ part of the sequence, and 7500A was used as the downstream primer to amplify the end 3′ part of the sequence (Yang et al., 2003). Sequencher software and online Oligonucleotide Properties Calculator^1^ were used to design primers for segmented amplification. Some primers were taken from a previous study by Han et al. (2020) (Supplementary Table S1). A QIAGEN One-Step RT-PCR kit was used (QIAGEN, Hilden, Germany). Amplification was performed using the following steps: reverse transcription (50°C, 30 min, 94°C, 2 min), sequence amplification (94°C, 30°C, 50°C, 30°C, 72°C, 1 min 20 s) for 40 cycles, treatment at 72°C for 7 min, and preservation at 4°C. PCR products were identified using 1% agarose gel electrophoresis and sent to Tsingke Biotechnology Co., Ltd. (Beijing, China) for sequence determination. The sequencing results were collated and spliced using Sequencher (v5.4.5) to obtain the final complete genome sequence.

### 2.4 Analysis of *VP1* sequences in CVA16 strains from Jixi City

MEGA7.0 software (v7.0.25) (Kumar et al., 2016) was used to verify the results of CVA16 genotyping. Nucleotide and amino acid genetic distances were calculated by “DISTANCE” program. The results of CVA16 genotyping were verified according to the division principle of 15–25% nucleotide difference of different genotypes within the same serotype proposed by Brown et al. (1999). Nucleotide and amino acid similarities of different gene subtypes of Jixi CVA16 strains were calculated using Sequence Identity Matrix program in BioEdit (v7.0.9.0) (Hall, 1999). ESPript3.0 online website was then used to analyze different Jixi CVA16 *VP1* gene subtype area locus mutations (Robert and Gouet, 2014). The PROVEAN program was used to evaluate whether the variations had an impact on the function of the VP1 protein. Use −2.5 as the reference threshold (a score ≤ − 2.5 is considered harmful compilation, and a score > − 2.5 is considered neutral variation) (Choi et al., 2012).

### 2.5 Phylogenetic analysis

To elucidate the global evolutionary origin of CVA16 B1c strains, complete CVA16 *VP1* sequences were obtained from GenBank and screened using TempEst software. Outliers were identified according to the vertical distance from the root to the node and residual, using the “root-to-tip” option to check the correlation (Rambaut et al., 2016). The “Models” package in MEGA software (v7.0.25) was used to select the optimal nucleotide replacement model. The molecular clock model was analyzed using a strict and an uncorrelated, relaxed clock combined with constant velocity, exponential growth, and logarithmic growth as the priority population growth modes. The Markov chain Monte Carlo method implemented in BEAST software (v1.10.4) (Suchard et al., 2018) was used, the chain length was set to 500,000,000, and the sampling frequency was set to 50,000. Convergence was tested using Tracer (v1.7.1) with an effective sample size > 200 as the criterion (Rambaut et al., 2018). A Bayesian maximum clade credibility (MCC) tree was constructed using TreeAnnotator (v1.10.4), and the top 10% of the sample trees were removed as burn-in. Phylogenetic trees were visualized using FigTree (v1.4.4). To analyze the dynamics of population diversity, Bayesian skyline plots were constructed based on the complete *VP1* sequences.

### 2.6 Evolutionary analysis

To determine the source of CVA16 B1c strains in China, the global dynamic propagation of CVA16 B1c viruses was analyzed. An asymmetric alternative model with the Bayesian stochastic search variable selection option was selected in BEAST to infer the spread rate of the possible regions of different nodes and to reconstruct the ancestral origin of all strains. SpreadD3 (Bielejec et al., 2016) was used to calculate migration paths and Bayesian factor values (BF) between different regions. The BF value was used as the criterion to determine possible paths. BF > 100 indicated extremely strong evidence support, 10 < BF ≤ 100 indicated strong evidence support, and 3 < BF ≤ 10 indicated moderate evidence support. The numbers of Markov jumps and rewards used for state transitions between different regions were used to describe the migration states entering and leaving a region (Minin and Suchard, 2008).

### 2.7 Recombination analysis

The whole genome sequences of the selected CVA16 B1c strains divided into the *5′-UTR*, *P1*, *P2,* and *P3* regions were compared with those of the CVA16 prototype strain. The EV-A prototype strain sequence was obtained from GenBank, and phylogenetic trees of CVA16 B1c and EV-A prototype strains were constructed based on the *5’-UTR*, *P1*, *P2,* and *P3* regions. BLAST was used to screen for other group A sequences with more than 85% similarity to each region of CVA16 B1c. Sequences from India, Malaysia, France, and China were randomly selected, along with one sequence from this study. Potential Recombination event analysis was conducted using SimPlot software (version 3.5.1) and Recombination Detection Program (RDP4, version 4.100). Seven methods from RDP4 were adopted, including RDP, GENECONV, 3Seq, Chimaera, SiScan, MaxChi and BootScan. Recombination detected by at least three of the seven methods was judged to be possible, with *p* ≤ 0.05 (Martin et al., 2015). The Kimura’s two-parameter model was adopted in Simplot for the nucleotide substitution model, and the ratio of nucleotide transition and transversion was set to 2.0 (Lole et al., 1999). The recombinant results were verified using BioEdit by comparing the similarity between the reference sequence, recombinant donor, and prototype strain in different segments.

## 3 Results

### 3.1 Pathogen spectrum composition in HFMD samples isolated in the Heilongjiang Province in 2022

**Figure 1:**
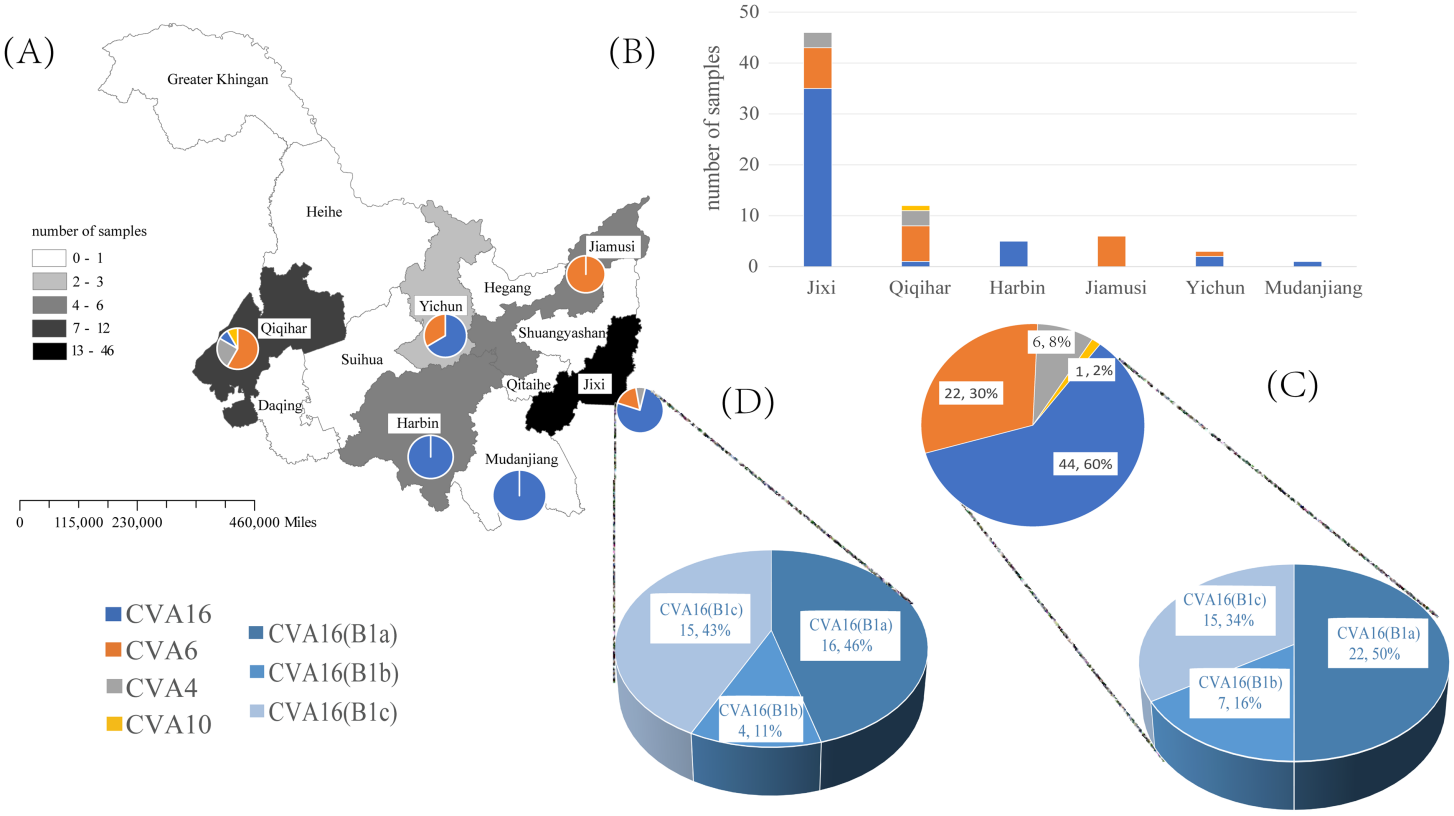
Spectrum of pathogenic coxsackievirus strains from HFMD samples isolated in the Heilongjiang Province (China) in 2022. **(A, B)** are graphs of the number of enterovirus pathogens detected in six regions of Heilongjiang Province. **(C)** represents the detected quantities of different enteroviruses and different subtypes of CVA16 in Heilongjiang Province; **(D)** represents the detected quantities of different subtypes of CVA16 in Jixi City.

**Figure 2:**
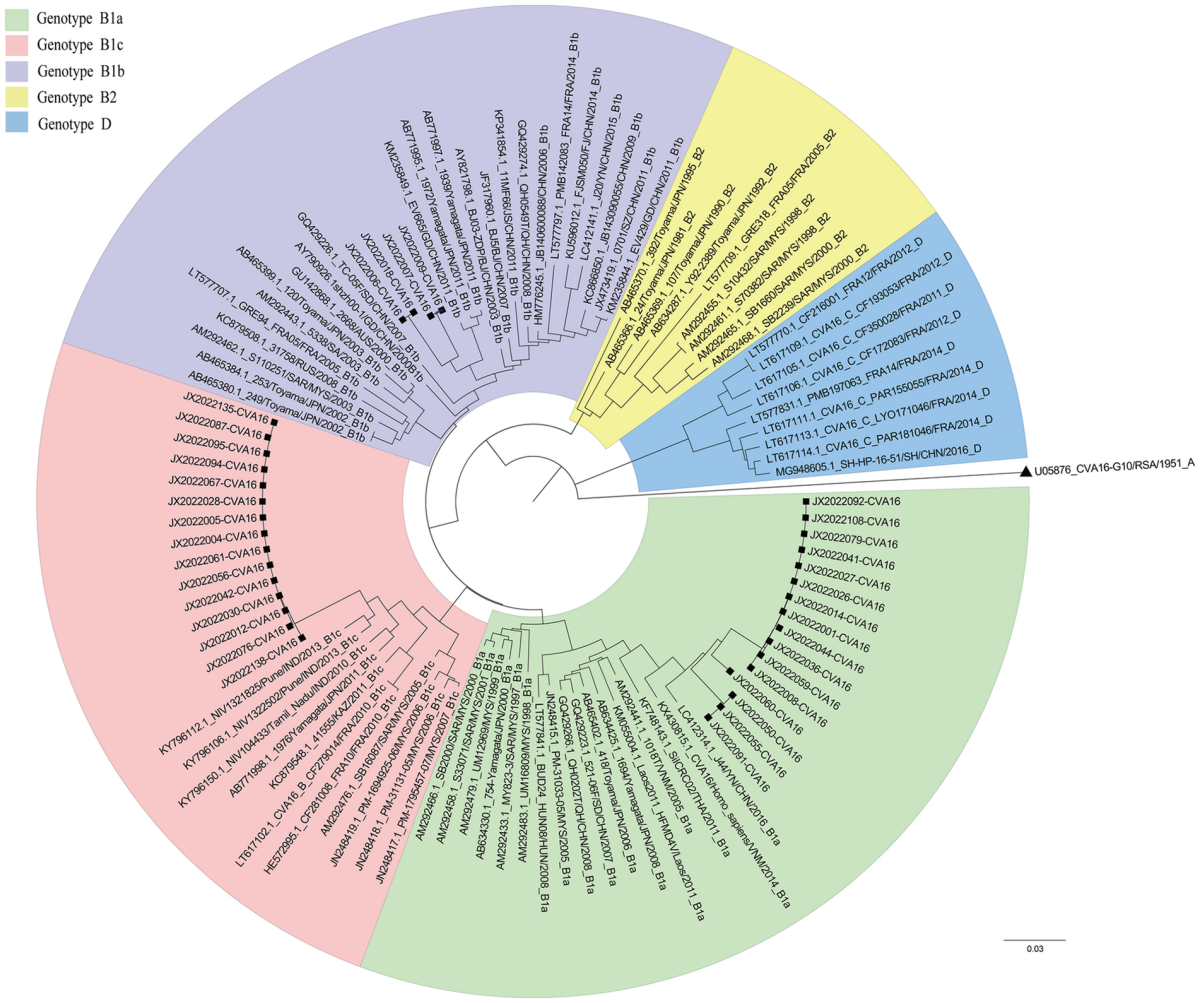
Phylogenetic tree of CVA16 strains from Jixi (Heilongjiang Province, China).

The *VP1* sequence was determined in 73 positive specimens from six cities in the Heilongjiang Province. There were significant differences in the pathogen spectrum composition (Figures 1A,B). CVA16 was mainly detected in Jixi, where most specimens were collected. The highest pathogen diversity was detected in Qiqihar. CVA16, CVA6, CVA4, and CVA10 strains were isolated there, with CVA6 strains being the predominant pathogens. In the present study, isolates detected in Harbin and Jiamusi were represented by a single subtype, CVA16 and CVA6, respectively. The lowest number of samples was sent from Mudanjiang, with only one CVA16 positive case. Overall, CVA16 strains were isolated from the largest number of detected cases, accounting for 60% (44 cases, mainly CVA16 B1a). CVA6 strains were second most prevalent (30%; Figure 1C). Further analysis of CVA16 positive samples from Jixi showed that 15 B1c strains were detected (Figure 2). This accounted for 43% of CVA16 samples from Jixi (Figure 1D). For more details, please refer to Supplementary Table S2.

### 3.2 Analysis of *VP1* gene characteristics of CVA16 strains from Jixi

The genetic distances between all genotypes were calculated and compared. The inter-genotype genetic distances were greater than 15%, whereas the intra-genotype distances were less than 15%. The genetic distances within each subtype of B genotype were 0.047–0.075. The genetic distances between the subtypes were 0.101–0.145, in the range of 8–15%. The nucleotide similarities of Jixi CVA16 B1c isolates were 99.6–100%. The nucleotide similarities between Jixi CVA16 B1c and Heilongjiang Province CVA16 B1a and CVA16 B1b were 86–88.5%. Four characteristic loci of amino acid mutations were revealed in CVA16 B1c and other clades in Jixi strains: P3S, V25A, I235V, and T240A (Supplementary Figure S1). The PROVEAN results showed that the PROVEAN scores of these four variations were 1.458, 0.064, −0.063 and 0.304 respectively, all belonging to neutral mutations.

### 3.3 Evolutionary dynamics of CVA16 B1c

**Figure 3:**
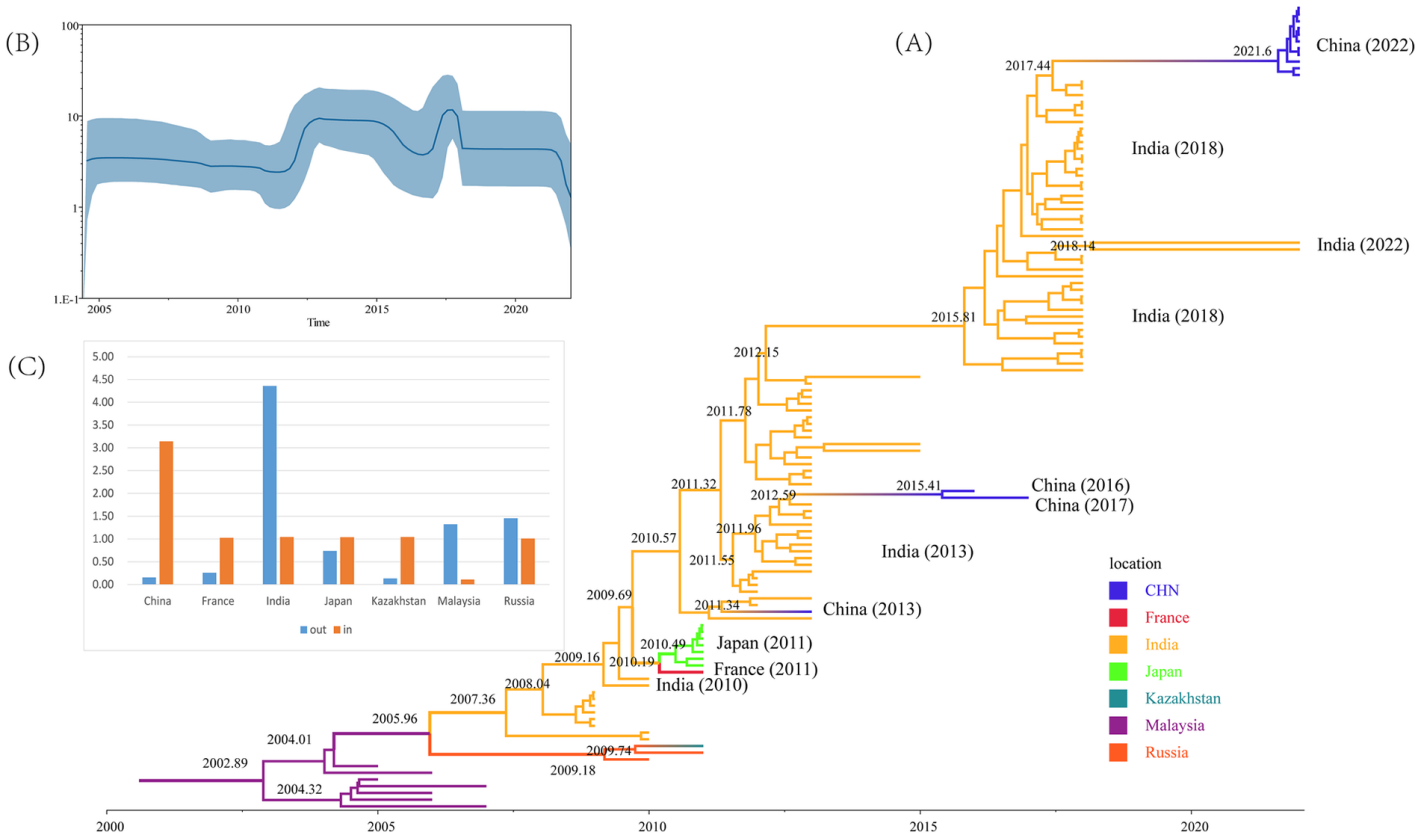
Evolutionary analyses of CVA16 B1c strains. **(A)** Maximum Clade Credibility tree built using the Markov chain Monte Carlo method for the global VP1 coding region. Branches are colored according to the country of isolation. **(B)** Bayesian skyline plot based on VP1 sequences of CVA16 B1c strains reflects the effective population size in 2004–2022. **(C)** Histograms based on Markov jumps and rewards in different countries.

In total, 120 CVA16 B1c sequences from 2005 to 2022 were screened by TempEst (Supplementary Table S3), and an uncorrelated relaxed clock and logarithmic prior by marginal likelihood value were selected as the best molecular clock model for CVA16 B1c to construct the MCC tree. The average base substitution rate of CVA16 B1c worldwide was estimated at 5.14 × 10^−3^ substitution/site/year with a 95% highest posterior density (HPD) of 4.13 × 10^−3^– 6.27 × 10^−3^ substitution/site/year. The most recent common ancestor (MRCA) of global CVA16 B1c was dated to 2003 (95% HPD: 2000–2004; Figure 3A). All Chinese strains clustered with Indian strains. Chinese strains from 2022 were closely related to the Indian strains from 2018, with an approximate earliest origin in 2021. Chinese strains isolated from 2013 to 2017 were closely related to the Indian strains isolated in 2013. The earliest strain, CVA16 B1c, isolated in China, was traced back to 2011 and was a Guangdong strain. No other related sequences were obtained during the same period, and this was presumed to be an imported case. All French and Japanese strains isolated in 2011 were most closely related to the Indian strains from 2010. It is likely that the Malaysian strains clustered into a single clade because they originated earlier. Bayesian skyline analysis showed that the effective population size changed over time (Figure 3B). It remained relatively stable until 2011, after which three fluctuations were observed. The first wave occurred in 2011–2012, with a small increase in the population. The second wave witnessed a dynamic change of decline, increase and then decline from 2015 to 2018, and then tended to stabilize. In the third wave of 2021, the population showed a downward trend again.

### 3.4 Analysis of the geographical spread of CVA16 B1c

**Figure 4:**
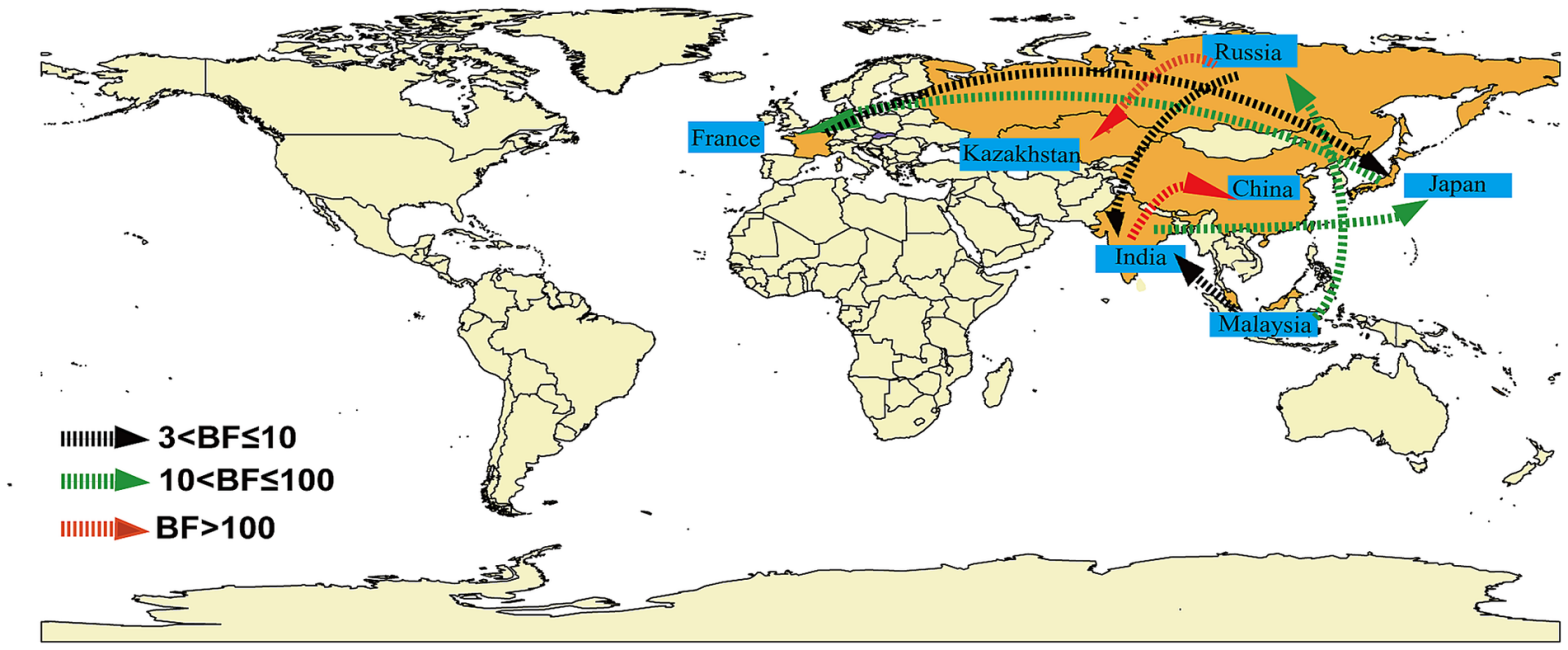
Analysis of the spatio-temporal dynamic propagation of CVA16 B1c strains. Only migration pathways with Bayesian factor (BF) values > 3 are shown. BF > 100 indicates extremely strong support, 10 < BF ≤ 100 indicates strong support, and 3 < BF ≤ 10 indicates moderate support.

Markov jumps and rewards analysis showed that India, Russia and Malaysia were mainly export-oriented, whereas other countries were mainly import-oriented (Figure 3C). To analyze the global dynamic propagation of CVA16 B1c further, the propagation network of CVA16 B1c strains was reconstructed. Geographical transmission analysis was performed for CVA16 B1c sequences from seven countries (14 from China, one from France, 88 from India, seven from Japan, one from Kazakhstan, seven from Malaysia, and two from Russia). Eight migration paths supported by BFs were identified (Figure 4). BF > 6,000 from India to China provided extremely strong support to the notion that Chinese CVA16 B1c strains were imported from India. The main migration paths could be: (I) from India to China, (II) from Russia to Kazakhstan, (III) from India to Japan, (IV) from Japan to France, (V) from Malaysia to Russia, (VI) from Malaysia to India, (VII) from Russia to India, and (VII) from France to Japan.

### 3.5 Recombination analysis

**Figure 5:**
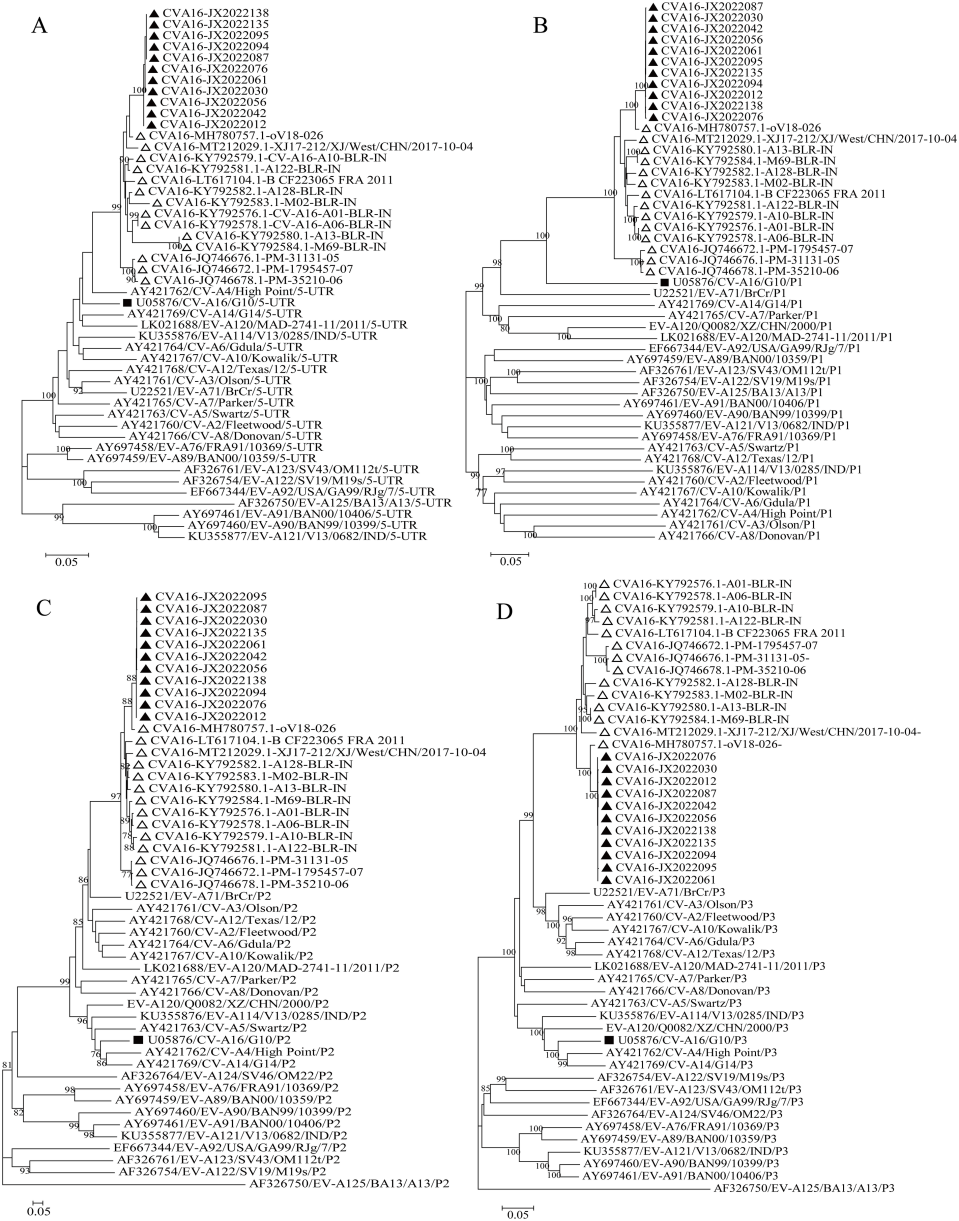
Phylogenetic trees based on the 5′-UTR, P1, P2, and P3 regions of CVA16 B1c genomes and prototype EV-A strains. **(A)** 5′-UTR; **(B)** P1 region; **(C)** P2 region; **(D)** P3 region. Notes: Triangles represent CVA16 B1c sequences. Black square represents the prototype strain.

**Figure 6:**
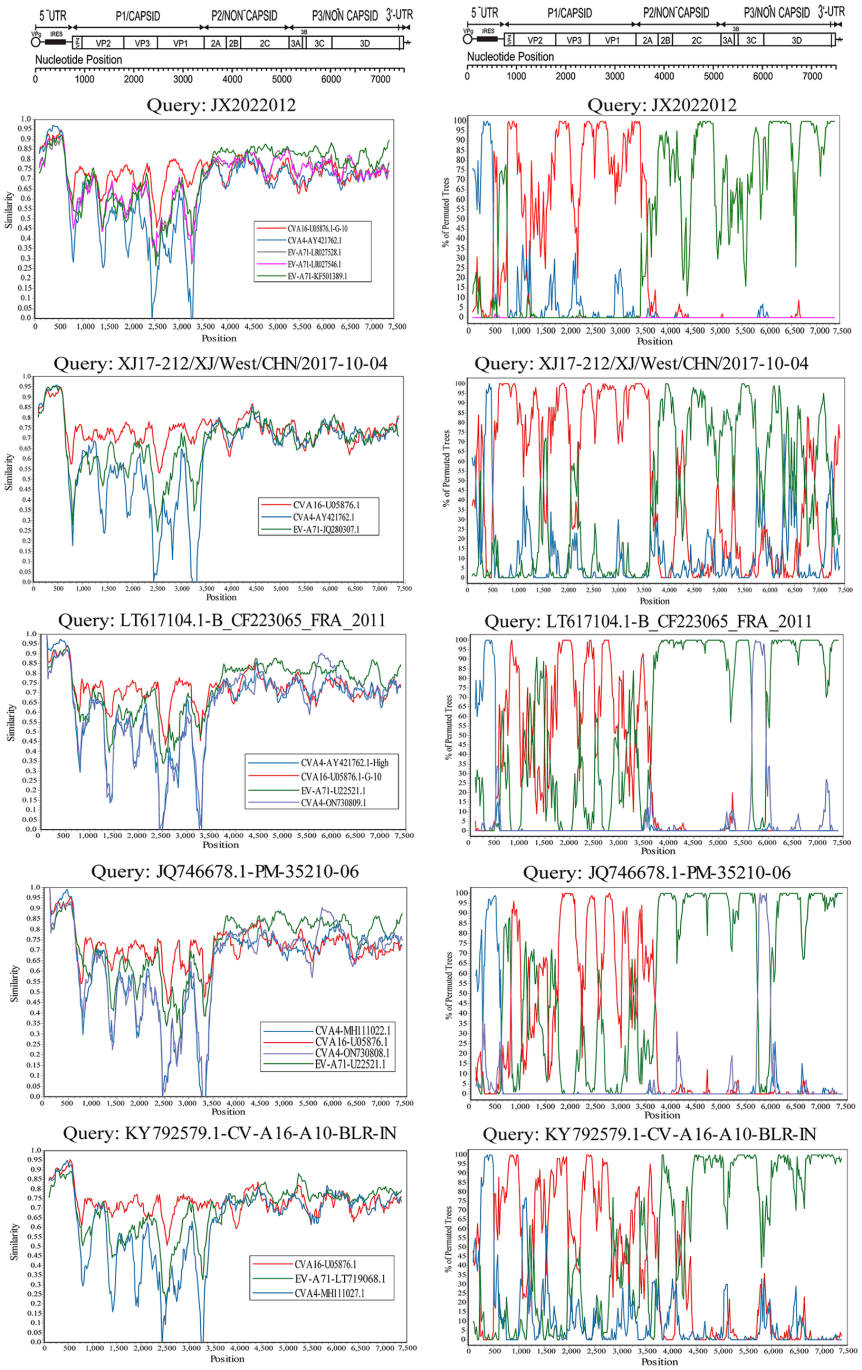
Bootscanning analysis of the whole CVA16 B1c strain genomes. The nucleotide transition and transversion ratios were set to 2.0, and the analysis was based on a 200-nucleotide sliding window with a 20-nucleotide step size.

The evolutionary trees of CVA16 5′-UTR, P1, P2, and P3 regions were constructed. The P1 region phylogenetic tree showed that all CVA16 B1c strains clustered together with CVA16 prototype strains (Figure 5B), which was consistent with the CVA16 serotyped results. However, phylogenetic trees based on the 5′-UTR, P2, and P3 regions showed different results. The CVA16 B1c strains clustered with CVA4 in the 5′-UTR region, and with EV-A71 in the P2 region (Figures 5A,C). The P3 region showed close similarity with EV-A71, CVA3, CVA12, CVA2, CVA6, and CVA10 (Figure 5D). Through RDP4 and Simplot analysis, it was found that the possible recombinant donors in the 5′-UTR region and the non-coding region were mainly CVA4 and EV-A71 (Figure 6 and Supplementary Figure S2). The similarity between different regions showed that the P1 region had the highest similarity with the CVA16 prototype strain. It has the highest similarity with EV-A71 in regions P2 and P3. The similarity with CVA4 was the highest in the 5’-UTR region (Supplementary Table S4).

## 4 Discussion

In this study, four characteristic loci were found in VP1 sequences of Jixi CVA16 B1c strains and strains of other clades: P3S, V25A, I235V, and T240A. Similarly, specific mutations at these sites were also found in the study of CVA16 B1c in Beijing and Guangdong (Hu et al., 2021; Liang et al., 2024; Zeng et al., 2025; Zhou et al., 2020). The VP1 region contains the major antigenic sites and most serotype-specific neutralization sites, and the substitution of amino acids may affect the function of proteins (Mateu, 1995). According to the prediction results of PROVEAN, P3S, V25A, I235V, and T240A are all neutral mutations. This may not cause a change in protein function, but it may also have an impact when it occurs simultaneously with the substitution of other regions. However, these mutations only exist in CVA16 B1c, and the same amino acid mutations have not been found in other genotypes of CVA16. At present, the impact of amino acid changes in B1c of CVA16 on the structure remains blank, and the biological significance reflected by such amino acid changes should be further evaluated. Whether these amino acid changes are related to virus antigenicity and pathogenicity needs further studies.

In the evolutionary analysis, the average nucleotide substitution rate of CVA16 B1c was estimated to be 5.14 × 10^−3^ substitution/site/year. Other studies on the evolutionary analysis of CVA16 have shown that the evolutionary rate of CVA16 is 3.36–6.66 × 10^−3^ substitution/site/year (Han et al., 2020; Zhao et al., 2016). It can be seen that CVA16 B1c does not show significant differences in terms of external evolutionary pressure. The analysis of global CVA16 B1c transmission suggested that Chinese B1c strains may have been imported from India. This conclusion is supported by a high Bayesian factor values. In this study, 15 CVA16 B1c strains were detected in the Heilongjiang Province of China, indicating a small outbreak. Apart from this study, no large-scale prevalence of B1c strains has been found in China, whereas long-term circulation of B1c strains have been reported in both India and Malaysia (Ganorkar et al., 2017; Chen et al., 2013; Chan et al., 2012). All CVA16 B1c strains in China were closely related to Indian strains, which further suggests the potential import risk of endemic strains in China and neighboring countries. Therefore, continuous and effective monitoring of HFMD worldwide should be emphasized to prevent the outbreak of CVA16 B1c in the future.

The symptoms of HFMD caused by CVA16 are generally mild: few severe cases and nervous system-related complications have been reported, and most of them were self-limiting (Zhu et al., 2010). However, some studies have shown that CVA16 can cause more serious complications and clinical symptoms (Wright et al., 1963; Wang et al., 2004), especially upon co-infection with EV-A71 (Chang et al., 1999; Cai et al., 2019). Recombination is one of the main mechanisms of enterovirus evolution. It was found in this study that CVA16 may have recombined with EV-A71 and CVA4. Other studies on CVA16 from Taiwan, Guangdong, central and northeastern China have suggested that the 5 ‘-UTR and non-structural protein coding regions of CVA16 subgenotypes B1 and B2 may recombine with CVA4 and EV-A71, respectively (Cheng et al., 2022; Han et al., 2014; Zhao et al., 2011; Lin et al., 2015). Previous studies have confirmed that a large outbreak of HFMD with fatal neurological complications in Fuyang City, China, in 2008 was mainly caused by the C4 sub-genotype of EV-A71, which had recombined with CVA16 in the 3D region (Zhang et al., 2010b). EV recombination may affect virulence and pathogenicity of the virus. Therefore, further attention should be paid to the recombination of CVA16 and EV-A71 strains.

Since the monovalent EV-A71 vaccine was launched in China the end of 2015, the detection rate of EV-A71 has decreased significantly. However, the lack of cross-protection against other EVs causing HFMD led to the detection rates of CVA6, CVA10 and CVA16 significantly exceeding that of EV-A71 (Zhang et al., 2022; Hong et al., 2022). At present, there is no specific vaccine for CVA16 to prevent HFMD on the market. Studies have indicated that there are EV-related multivalent vaccines in the clinical trial stage. These multivalent vaccines contain CVA16 and are developed based on the previously prevalent B1a and B1b subtypes (Zeng et al., 2025). CVA16 B1c has been occasionally detected in some provinces in recent years, such as Shanghai (Wang et al., 2018b), Beijing (Hu et al., 2021; Liang et al., 2024), and Guangdong (Zeng et al., 2025). Due to the antigenic differences among subgenotypes, it is necessary to develop an effective multivalent vaccine with cross-protective immune response. In addition to vaccines, disease prevention and control also requires further strengthening of comprehensive monitoring of EVs. The Asia-Pacific Network for Enterovirus Surveillance (APNES) was established in 2017 to assess the burden of disease through laboratory diagnosis and data collection (Chiu et al., 2020). The European Union has also established the European non-polio enterovirus network (ENPEN) to improve the diagnosis of enteroviruses, monitor enterovirus data and prevent the spread of enteroviruses (Harvala et al., 2018). For China, merely conducting HFMD monitoring cannot detect all enteroviruses in a timely manner. To supplement its limitations, a network of encephalitis and meningitis monitoring laboratories was established based on sentinel hospitals. At the same time, patients with acute flaccid paralysis (AFP) were monitored, and environmental surveillance of sewage water was carried out. Strengthen prevention and control measures to reduce the risk of epidemic outbreak.

In conclusion, this study is the first detection of CVA16 B1c in Heilongjiang province. Combined with the available CVA16 B1c data in the GenBank database, the isolated CVA16 B1c in Jixi City was analyzed. The global phylogeny and spread of CVA16 B1c were deeply understood through the Bayesian method, and recombination analysis was carried out. This study enriched the global genetic database of CVA16 B1c and provided an epidemiological basis for its further research.

## Data Availability

The accession numbers NMDCN0007NAQ - NMDCN0007NB8 of the National Microbiology Data Center (NMDC).

## Footnotes

^1^ https://www.northwestern.edu/
