# Supplementary material for: Characteristics of CVA16 B1c strains isolated for the first time in the Heilongjiang Province of China
Source: Front Microbiol. 2025 Aug 6;16:1634547. doi: 10.3389/fmicb.2025.1634547 (PMC12364893; doi:10.3389/fmicb.2025.1634547)
Supplement: Supplementary file 1 [file Data_Sheet_1.docx]

Supplementary Materials

**Table S1. Primers used for the full-length genomic sequencing of CVA16 B1c.**

| Primer | Nucleotide position(nt) | Sequence（5’-3’） | Orientation | Reference |
| --- | --- | --- | --- | --- |
| 0001S48 |  | GGGGACAAGTTTGTACAAAAAAGCAGGCTTT | Forward | [29] |
| 265R | 246-265 | GGTTTCCCGAAGTAGTTAGCC | Reverse | This study |
| 65F | 65-84 | CGGAATCTTTGTGCGCCTGT | Forward | This study |
| 569R | 544-569 | ACACGGACACCCAAAGTAGTCGGTTC | Reverse | This study |
| 1125F | 1125-1148 | GATGCAACRGCAGTCGACAARCCC | Forward | [20] |
| 2095R | 2072-2095 | CYGCAAACATGAATGTYACCTCCA | Reverse | [20] |
| 1820F | 1820-1841 | CTCCGCCTATACACATACCAG | Forward | This study |
| 2702R | 2683-2702 | TTCCCAATGGCTGTCTCCTG | Reverse | This study |
| 2344F | 2344-2367 | ATTGGTGCTCCYACTACAGCRTAT | Forward | [20] |
| 3345R | 3322-3345 | CARCGTTGTTATCTTGTCTCTRCT | Reverse | [20] |
| 3215F | 3215-3235 | TGAGGATTAAACACGTYAGGG | Forward | This study |
| 4461R | 4441-4461 | GGTTCAATACGGTGTTTGCTC | Reverse | This study |
| 4342F | 4342-4365 | TGTTTGGYAATGTGTCATATTTRG | Forward | [20] |
| 5389Y | 5366-5389 | GCTCGGTCCTTGRACYGTGGCTGT | Reverse | [20] |
| 5204Y | 5204-5223 | CCAGAAACACCGACCAATGT | Forward | [20] |
| 6157Z | 6138-6157 | GTCAGGCTCGTGTAGGGTGT | Reverse | [20] |
| 5994F | 5994-6015 | CTGGGAGGCTTAACATTAATGG | Forward | This study |
| 6857R | 6837-6857 | GGAGTTGAAAATGGAAGTGCC | Reverse | This study |
| 6663F | 6663-6682 | TCAGGATATGACGCGAGCCT | Forward | This study |
| 7033R | 7014-7033 | GCGGGTGTCATTGTCAADCC | Reverse | This study |
| 6907F | 6907-6926 | GGATCGATTTGGATGAGTTG | Forward | This study |
| 7500A |  | GGGGACCACTTTGTACAAGAAAGCTGGG(T)24 | Reverse | [29] |

**Table S2. Information about the 15 strains of CVA16 B1c in this study.**

| **Strain name** | **Separate area** | **Sampling date** | **Specimen type** | **Isolated population** | **Case type** | **genotyping** | **NMDC accession numbers** |
| --- | --- | --- | --- | --- | --- | --- | --- |
| JX2022004 | Jixi Heilongjiang | 2022.07.25 | Throat swab | HFMD case | Mild | CVA16(B1c) | NMDCN0007NB8 |
| JX2022005 | Jixi Heilongjiang | 2022.07.13 | Throat swab | HFMD case | Mild | CVA16(B1c) | NMDCN0007NB7 |
| JX2022012 | Jixi Heilongjiang | 2022.07.19 | Throat swab | HFMD case | Mild | CVA16(B1c) | NMDCN0007NB6 |
| JX2022028 | Jixi Heilongjiang | 2022.08.02 | Throat swab | HFMD case | Mild | CVA16(B1c) | NMDCN0007NB5 |
| JX2022030 | Jixi Heilongjiang | 2022.08.09 | Throat swab | HFMD case | Mild | CVA16(B1c) | NMDCN0007NB4 |
| JX2022042 | Jixi Heilongjiang | 2022.09.27 | Throat swab | HFMD case | Mild | CVA16(B1c) | NMDCN0007NB3 |
| JX2022056 | Jixi Heilongjiang | 2022.10.04 | Throat swab | HFMD case | Mild | CVA16(B1c) | NMDCN0007NB2 |
| JX2022061 | Jixi Heilongjiang | 2022.10.08 | Throat swab | HFMD case | Mild | CVA16(B1c) | NMDCN0007NB1 |
| JX2022067 | Jixi Heilongjiang | 2022.10.11 | Throat swab | HFMD case | Mild | CVA16(B1c) | NMDCN0007NB0 |
| JX2022076 | Jixi Heilongjiang | 2022.10.25 | Throat swab | HFMD case | Mild | CVA16(B1c) | NMDCN0007NAV |
| JX2022087 | Jixi Heilongjiang | 2022.10.10 | Throat swab | HFMD case | Mild | CVA16(B1c) | NMDCN0007NAU |
| JX2022094 | Jixi Heilongjiang | 2022.10.30 | Throat swab | HFMD case | Mild | CVA16(B1c) | NMDCN0007NAT |
| JX2022095 | Jixi Heilongjiang | 2022.10.29 | Throat swab | HFMD case | Mild | CVA16(B1c) | NMDCN0007NAS |
| JX2022135 | Jixi Heilongjiang | 2022.11.09 | Throat swab | HFMD case | Mild | CVA16(B1c) | NMDCN0007NAR |
| JX2022138 | Jixi Heilongjiang | 2022.11.17 | Throat swab | HFMD case | Mild | CVA16(B1c) | NMDCN0007NAQ |

**Table S3 GenBank accession numbers for the CVA16 B1c sequences involved in this study.**

| GenBank number | Sequence name | isolated country | Separation time |
| --- | --- | --- | --- |
| AB771973.1 | 1716 | Japan | 2011 |
| AB771984.1 | 1812 | Japan | 2011 |
| AB771985.1 | 1822 | Japan | 2011 |
| AB771998.1 | 1976 | Japan | 2011 |
| AB771999.1 | 1993 | Japan | 2011 |
| AB772003.1 | 2163 | Japan | 2011 |
| AB772007.1 | 2680 | Japan | 2011 |
| AM292476.1 | SB16087-SAR-05 | Malaysia | 2005 |
| JN248419.1 | PM-1694925-06 | Malaysia | 2006 |
| JN248422.1 | PM-1824818-07 | Malaysia | 2007 |
| JQ746668.1 | PM-1651402-06 | Malaysia | 2006 |
| JQ746672.1* | PM-1795457-07 | Malaysia | 2007 |
| JQ746676.1* | PM-31131-05 | Malaysia | 2005 |
| JQ746678.1* | PM-35210-06 | Malaysia | 2006 |
| KC879526.1 | 38394_CVA16_RUS_2010 | Russia | 2010 |
| KC879548.1 | 41555_CVA16_KAZ_2011 | Kazakhstan | 2011 |
| KC879554.1 | 42234_CVA16_RUS_2011 | Russia | 2011 |
| KM235916.1 | EV1506 | CHN | 2013 |
| KY792576.1* | CV-A16-A01-BLR-IN | India | 2012 |
| KY792578.1* | CV-A16-A06-BLR-IN | India | 2013 |
| KY792579.1* | CV-A16-A10-BLR-IN | India | 2013 |
| KY792580.1* | CV-A16-A13-BLR-IN | India | 2013 |
| KY792581.1* | CV-A16-A122-BLR-IN | India | 2015 |
| KY792582.1* | CV-A16-A128-BLR-IN | India | 2015 |
| KY792583.1* | CV-A16-M02-BLR-IN | India | 2013 |
| KY792584.1* | CV-A16-M69-BLR-IN | India | 2015 |
| KY796107.1 | NIV1321823 | India | 2013 |
| KY796108.1 | NIV1322107 | India | 2013 |
| KY796109.1 | NIV1322374 | India | 2013 |
| KY796110.1 | NIV1322102 | India | 2013 |
| KY796111.1 | NIV1321420 | India | 2013 |
| KY796112.1 | NIV1321825 | India | 2013 |
| KY796113.1 | NIV1322365 | India | 2013 |
| KY796114.1 | NIV1321819 | India | 2013 |
| KY796115.1 | NIV1321433 | India | 2013 |
| KY796117.1 | NIV1320588 | India | 2013 |
| KY796118.1 | NIV1320913 | India | 2013 |
| KY796119.1 | NIV1321824 | India | 2013 |
| KY796120.1 | NIV1322368 | India | 2013 |
| KY796122.1 | NIV1323507 | India | 2013 |
| KY796123.1 | NIV1323513 | India | 2013 |
| KY796124.1 | NIV1324322 | India | 2013 |
| KY796125.1 | NIV1321821 | India | 2013 |
| KY796127.1 | NIV1323499 | India | 2013 |
| KY796128.1 | NIV1322716 | India | 2013 |
| KY796131.1 | NIV1320907 | India | 2013 |
| KY796132.1 | NIV1321466 | India | 2013 |
| KY796133.1 | NIV1321460 | India | 2013 |
| KY796134.1 | NIV1321436 | India | 2013 |
| KY796139.1 | NIV1211492 | India | 2012 |
| KY796141.1 | NIV1211445 | India | 2012 |
| KY796142.1 | NIV0916024 | India | 2009 |
| KY796144.1 | NIV0916022 | India | 2009 |
| KY796145.1 | NIV0924312 | India | 2009 |
| KY796146.1 | NIV0924311 | India | 2009 |
| KY796147.1 | NIV0916021 | India | 2009 |
| KY796148.1 | NIV0915920 | India | 2009 |
| KY796149.1 | NIV0104441 | India | 2010 |
| KY796153.1 | NIV104445 | India | 2010 |
| KY796154.1 | NIV1050768 | India | 2010 |
| KY796155.1 | NIV1050767 | India | 2010 |
| LT617104.1* | CVA16_B_CF223065_FRA_2011 | France | 2011 |
| MF094720.1 | NIV1211473 | India | 2012 |
| MH780757.1* | oV18-026 | India | 2018 |
| MH796400.1 | V18-022 | India | 2018 |
| MT212029.1* | XJ17-212 | CHN | 2017 |
| MT553178.1 | S3540 | CHN | 2016 |
| MT577654.1 | NIV1850130 | India | 2018 |
| MT577655.1 | NIV1850166 | India | 2018 |
| MT577656.1 | NIV1850125 | India | 2018 |
| MT577657.1 | NIV1850158 | India | 2018 |
| MT577658.1 | NIV1850124 | India | 2018 |
| MT577659.1 | NIV1850123 | India | 2018 |
| MT577660.1 | NIV1850113 | India | 2018 |
| MT577661.1 | NIV1850112 | India | 2018 |
| MT577662.1 | NIV1850111 | India | 2018 |
| MT577663.1 | NIV1850164 | India | 2018 |
| MT577664.1 | NIV1850163 | India | 2018 |
| MT577665.1 | NIV1850153 | India | 2018 |
| MT577666.1 | NIV1850155 | India | 2018 |
| MT577667.1 | NIV1850154 | India | 2018 |
| MT577668.1 | NIV1850132 | India | 2018 |
| MT577669.1 | NIV1850140 | India | 2018 |
| MT577670.1 | NIV1850084 | India | 2018 |
| MT577671.1 | NIV1850085 | India | 2018 |
| MT577672.1 | NIV1850086 | India | 2018 |
| MT577673.1 | NIV1841810 | India | 2018 |
| MT577674.1 | NIV1841809 | India | 2018 |
| MT577675.1 | NIV1850087 | India | 2018 |
| MT577676.1 | NIV1850170 | India | 2018 |
| MT577677.1 | NIV1850173 | India | 2018 |
| MT577678.1 | NIV1850169 | India | 2018 |
| MT577679.1 | NIV1850167 | India | 2018 |
| MT577680.1 | NIV1850127 | India | 2018 |
| MT577681.1 | NIV1850142 | India | 2018 |
| MT577682.1 | NIV1850143 | India | 2018 |
| MT577683.1 | NIV1850114 | India | 2018 |
| MT577684.1 | NIV1841374 | India | 2018 |
| MT577685.1 | NIV1850180 | India | 2018 |
| MT577686.1 | NIV1850097 | India | 2018 |
| MT577687.1 | NIV1850102 | India | 2018 |
| MT577688.1 | NIV1850103 | India | 2018 |
| MT577689.1 | NIV1850148 | India | 2018 |
| MT577690.1 | NIV1850150 | India | 2018 |
| MT577691.1 | NIV1850151 | India | 2018 |
| MT577693.1 | NIV1850177 | India | 2018 |
| MT577694.1 | NIV1850147 | India | 2018 |
| OR437338.1* | CV-A16/123/SST/NIV/IND/2022 | India | 2022 |
| OR750554.1* | A16/AIIMS/Microbiology/2022 | India | 2022 |

* Represents the whole genome sequence of CVA16 (B1c) in GenBank.

**Table S4 Nucleotide similarity between CVA16 B1c reference sequence, prototype strain and recombinant donor.**

| name | Recombinant donor | 5'UTR | P1 | P2 | P3 |
| --- | --- | --- | --- | --- | --- |
| JX2022012 (2022) | CVA4 | 86.8% | 62.9% | 78.3% | 77.6% |
|  | CVA16 | 85.6% | 75.8% | 79.9% | 78.0% |
|  | EV-A71 | 79.4%-84.3% | 61.4%-69.2% | 81%-84% | 79.5%-83.7% |
| MT212029.1-XJ17-212/XJ/West/CHN/2017-10-04 (2017) | CVA4 | 86.6% | 62.6% | 78.3% | 77.1% |
|  | CVA16 | 85.6% | 75.9% | 79.7% | 77.6% |
|  | EV-A71 | 82.9%-86.3% | 68.3%-69.2% | 79.1%-83.6% | 77.6%-83.7% |
| LT617104.1-B_CF223065_FRA_2011 (2011) | CVA4 | 68.9%-87.5% | 62.5%-62.7% | 77.6%-77.9% | 77.4%-79.1% |
|  | CVA16 | 86.0% | 76.0% | 79.1% | 77.4% |
|  | EV-A71 | 83.6% | 69.4% | 83.3% | 82.5% |
| JQ746678.1-PM-35210-06 (2006) | CVA4 | 71.3%-85.5% | 62.3%-62.7% | 78%-78.7% | 78.3%-79.4% |
|  | CVA16 | 84.6% | 75.0% | 79.5% | 77.2% |
|  | EV-A71 | 82.7% | 69.6% | 83.8% | 83.3% |
| KY792579.1-CV-A16-A10-BLR-IN (2013) | CVA4 | 82.8%-87% | 62%-62.3% | 76%-77.1% | 76.5%-78% |
|  | CVA16 | 86.1% | 75.6% | 77.9% | 77.9% |
|  | EV-A71 | 83.1% | 68.7% | 79.0% | 80.7% |

**Figure S1. Amino acid mutation in the VP1 region of CVA16 from Jixi**

**
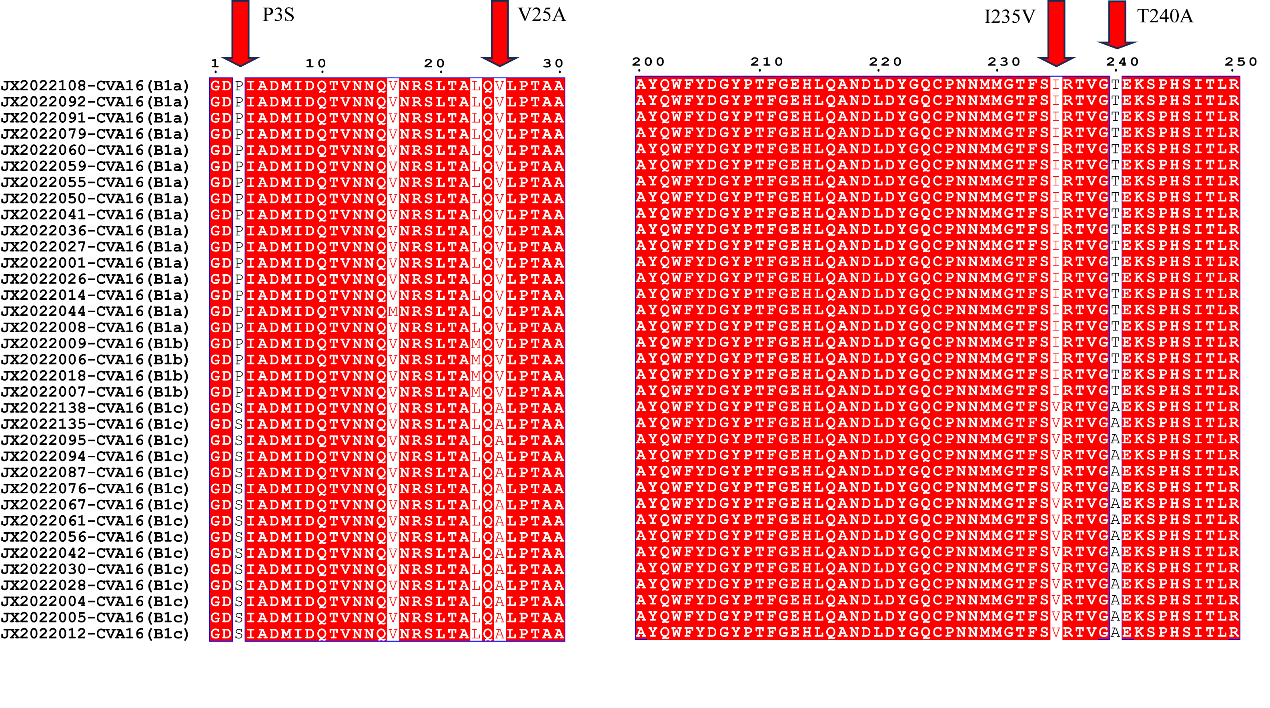
**

**Figure S2.** **The genomic map of CVA16 B1c recombination events predicted by RDP4. The black band represents the full-length genome of CVA16 B1c; the number above indicates beginning and ending breakpoint positions. The grey band represents the genomic region where recombination events may occur.**

**
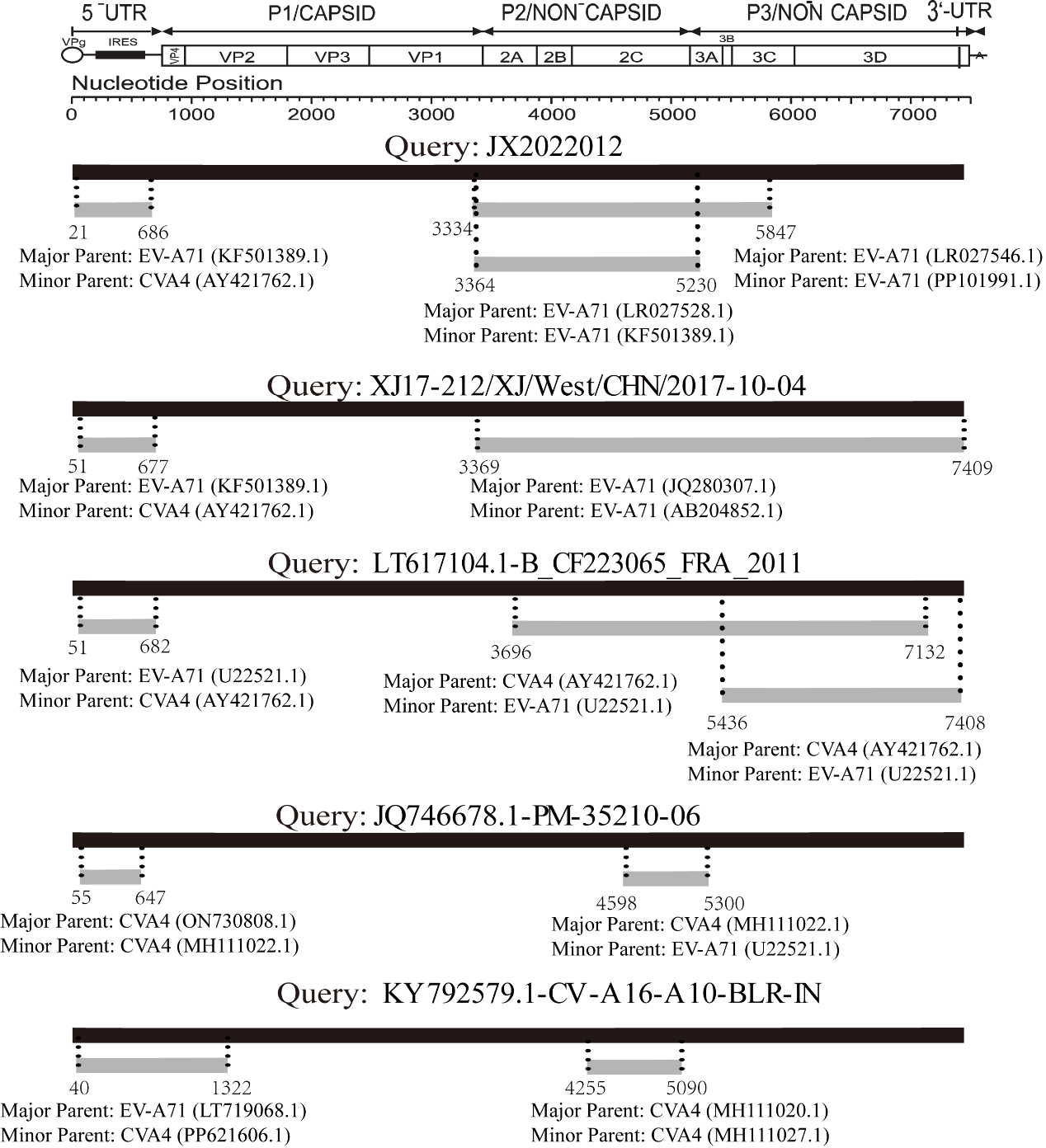
**
